# Structured reviews pilot in *mSphere*

**DOI:** 10.1128/msphere.00847-24

**Published:** 2024-10-28

**Authors:** Michael J. Imperiale

**Affiliations:** 1 University of Michigan, Ann Arbor, Michigan, USA

## EDITORIAL

On 6 November 2024, *mSphere* will initiate a 6-month pilot in which we will solicit structured reviews of manuscripts. The purpose of the structured review is to provide the reviewer with better guidance about which aspects of the manuscript ought to be clearly addressed. For example, the review form will ask specific questions about the clarity of the writing and figures, the use of appropriate controls, and whether the literature is appropriately cited. We have posted on our webpage (https://journals.asm.org/journal/msphere/submission-review-process) a list of questions that will be found on the new review form. The reviewer will be asked to reply to these questions and will still provide a typical narrative review. As such, the structured part of the review form provides not only a framework with which to think about the review but also a means for the reviewer to double check that critical components of a manuscript are commented upon in the narrative.

We think that this approach will be beneficial to all our stakeholders. Reviewers, especially those who are new to the process, will have a clear indication of what characteristics of the manuscript they ought to focus on. Editors will have uniform and clearer information from which to make decisions. Authors, in turn, will have in the reviews an unambiguous indication of which items need either more experimental work or better writing.

We are by no means pioneers in this effort: other journals have already adopted this approach, with excellent success (e.g., https://www.nature.com/articles/d41586-024-01101-9 and https://peerj.com/articles/17514/). We ourselves will be conducting surveys to assess whether this format is helpful to the *mSphere* community. The pilot will run for 6 months, at which point we will review the feedback and decide whether to continue. We would very much appreciate hearing from you, either via the survey or independently by emailing msphere@asmusa.org.

## AFTER EPUB

*[This article was published on 28 October 2024 with a date error in the text. The date was corrected in the current version, posted on 30 October 2024.]*

